# Propagating, Evanescent, and Complex Wavenumber Guided Waves in High-Performance Composites

**DOI:** 10.3390/ma12020269

**Published:** 2019-01-15

**Authors:** Victor Giurgiutiu, Mohammad Faisal Haider

**Affiliations:** Department of Mechanical Engineering, University of South Carolina, Columbia, SC 29208, USA; victorg@mailbox.sc.edu

**Keywords:** wave propagation, real wavenumbers, imaginary wavenumbers, complex wavenumber, Rayleigh-Lamb equation, semi-analytical finite element method, anisotropic materials, CFRP

## Abstract

The study of propagating, evanescent and complex wavenumbers of guided waves (GWs) in high-performance composites using a stable and robust semi-analytical finite element (SAFE) method is presented. To facilitate understanding of the wavenumber trajectories, an incremental material change study is performed moving gradually from isotropic aluminum alloy to carbon fiber reinforced polymer (CFRP) composites. The SAFE results for an isotropic aluminum alloy plate are compared with the exact analytical solutions, which shows that N = 20 SAFE elements across the thickness provides <0.5% error in the highest evanescent wavenumber for the given frequency-wavenumber range. The material change study reveals that reducing the transverse and shear moduli moves the wavenumber solution towards one similar to composite material. The comparison of the propagating, evanescent and complex wavenumber trajectories between composites and aluminum alloy show that antisymmetric imaginary Lamb wave modes always exist in composites although they may not exist in isotropic aluminum alloy at some frequencies. The wavenumber trajectories for a unidirectional CFRP plate show that the range of real wavenumber is much smaller than in the isotropic aluminum alloy. For laminated CFRP composite plates (e.g., unidirectional, off-axis, transverse, cross-ply and quasi-isotropic laminates), the quasi Lamb wave and shear horizontal (SH) wave trajectories are also identified and discussed. The imaginary SH wave trajectories in laminated composites are distorted due to the presence of ±45 plies. The convergence study of the SAFE method in various CFRP laminates indicates that sufficient accuracy can always be achieved by increasing the number of SAFE elements. Future work will address the stress-continuity between composite layers.

## 1. Introduction

The guided wave damage detection technique is popular in the nondestructive evaluation (NDE) and structural health monitoring (SHM) community for observing the evolution of the material state in a safety-critical structure. Guided waves (GWs) are waves propagating in a wave guide while being confined by the material boundary or by the boundaries separating different materials. A typical case is that of GWs traveling in a plate which is bounded by its top and bottom faces; thus, GWs propagate along the plate and parallel to top and bottom plate boundaries. The GWs are created by interactions of the bulk waves with the boundaries. These interactions create standing wave modes across the plate thickness; these standing modes propagate along the plate being constrained by the plate boundaries. A straight-crested guided wave propagating in a plate has the generic expression:

(1) $$\Phi (x,y,t)=f(y)e^{i(\xi x-\omega t)}$$

where x is the wave propagation direction, f(y) describes the thickness-wise variation, ω is the circular frequency, and ξ is the wavenumber related to the wavespeed c by the relation ξ=ω/c. Imposition of stress-free boundary conditions at the top and bottom faces of the plate yields a characteristic equation that depends on frequency ω and wavenumber ξ. For a given ω, one solves numerically the characteristic equation to find the corresponding ξ. It turns out that the number of roots of the characteristic equation is infinite; for each root $\xi_{n}$, n=0,1,… there will be a specific function $f_{n}(y)$, usually called "modeshape". If the frequency is allowed to vary over a prescribed interval, then wavenumber families appear; these are usually called dispersion curves.

The roots of the characteristic equation lie in the complex domain, ξ∈ℂ, with some being found on the real axis, ξ∈ℝ, others on the imaginary axis ξ∈I, while many are just complex, ξ∈ℂ. The real wavenumbers ξ∈ℝ correspond to propagating waves, whereas the imaginary wavenumbers ξ∈I correspond to so-called evanescent waves that do not propagate but represent a local vibration fast decaying away from the source. The complex wavenumbers ξ∈ℂ represent propagating waves that are fast decaying away from the source. The imaginary and complex wavenumbers occur in complex-conjugate pairs, such that spatial decay is ensured in both positive and negative *x* directions.

For isotropic materials, e.g., metallic plates, the analytical prediction of guided-wave dispersion curves is well developed in the classical works of Rayleigh [1], Lamb [2], Viktorov [3], and others. In isotropic plates, the characteristic equation is known as the Rayleigh-Lamb equation. Much of the previous work has been focused on real wavenumbers ξ∈ℝ, since these are of interest for practical damage detection applications utilizing propagating ultrasonic waves. 

**Figure 1:**
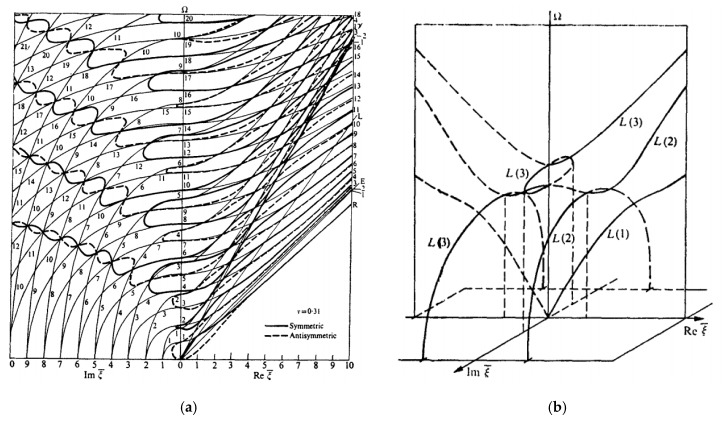
Frequency-wave number solution of Rayleigh-Lamb equations for mixed boundary in an isotropic plate (υ=0.31) (**a**) Real and imaginary wavenumber dispersion curves (**b**) 3D representation of the complex-wavenumber trajectories for the first three longitudinal modes [10].

However, the imaginary ξ∈I and complex ξ∈ℂ wavenumbers become of interest when studying how guided wave interact with damage or discontinuity in a structure. The scattering of GWs from any kind of damage is a complex phenomenon involving propagating and evanescent, and complex guided wave modes [4,5,6]. The evanescent and complex GWs exist only near the source. The damage acts as a secondary wave source. Therefore, for considering scattering of GWs by damage, one has to consider all wave types, both propagating and nonpropagating, to meet the power flow balanced at the damage location. Wave-damage interaction results in GW reflection, transmission, and mode conversion. In such analysis, normal mode expansion utilizing a combination of propagating, evanescent, and complex-wavenumber modes was found to be both highly efficient and numerically exact [4,7,8]. The rationale is that in the near vicinity of the damage, the evanescent and complex-wavenumber GWs are still present and play an active role in the satisfaction of the local boundary conditions on the damaged edge. Poddar et al. [4] developed a unique algorithm to obtain the complex wavenumber solution of guided wave equations in an isotropic plate. A brief description of how this method extracts the real, imaginary, and complex wavenumbers of guide waves in an isotropic plate is presented in Appendix A. The complex modes corresponding to complex wave numbers have been used to solve the Lamb wave scattering problem from discontinuity or damage of a structure. Hence, in this case, the dispersion curves for imaginary and complex wavenumbers are only those needed as for real wavenumbers. Fortunately, the study of imaginary and complex wavenumber dispersion curves in isotropic materials is well documented (Mindlin [9] and Graff [10]). For illustration, Figure 1 reproduces from reference [10] the plot of wavenumber roots for mixed boundary conditions in an isotropic plate with Poisson’s ratio υ=0.31. Figure 1a shows the real and imaginary wavenumber trajectories, whereas Figure 1b shows a 3D representation of the complex-wavenumber trajectories for the first, second, and third longitudinal modes.

High-performance composites are popular in many applications such as aerospace, automotive, defense, marine and other structural applications [11,12,13,14,15]. Composites are heterogeneous materials with anisotropic properties. High-performance composites consist of multiple layers with different orientation. Their anisotropy and layered construction present a challenge for the calculation of guided wave dispersion curves and associate modeshapes. The practical use of guided-wave principles to detect damage in composite structures has mostly focused on propagating-wave principles, and the analytical work on guided-wave in high-performance composites has been focused on real wavenumbers ξ∈ℝ with the subsequent finding of dispersion curves and corresponding modeshapes.

Many researchers have reported the experimentally-observed sensitivity of guided waves upon interacting with different types of damage [16,17,18,19,20]. The modeling of this wave-damage interaction in composite materials has only been possible, so far, through the finite element method (FEM), which requires extensive computational resources for a correct convergence at ultrasonic frequencies. In contrast, analytical models, if they existed, would be computationally efficient and would allow extensive parametric studies to identify optimal SHM system characteristics. Analytical models of wave-damage interaction in composites similar to those obtained in isotropic materials [4] have not yet been reported, although their potential utility is unquestionable. As the research into analytical modeling of the interaction between GWs and damage in composite materials progresses, the importance of understanding the behavior of imaginary and complex wavenumber in composite materials grows. However, the study of the imaginary and complex wavenumbers for GWs in composite materials is not as well documented in the literature as it is for isotropic metallic plates [9,10].

The three different groups of GWs corresponding to real, imaginary, and complex wavenumbers have very different physical behavior despite being solutions of the same governing equation. The propagating wave corresponding to the real wavenumber solution may change due to changes of the material properties or damage of the composite. In such cases, imaginary and complex wavenumber dispersion curve could be useful to identify a change in the material state of the composite. Therefore, weak adhesive bonding between layers and delamination type damage would create different dispersion phenomena than for the pristine structure.

The availability of the complete (real, imaginary and complex) wavenumber solution for GWs in composite materials opens the road for developing an analytical framework for studying the interaction of guides waves with damage/discontinuities in composite structures using a normal-mode expansion (NME) approach. The guided-wave scattering process is a complex phenomenon involving propagating, evanescent and complex guided wave modes. Although only the propagating modes travel away from the damage as scattered waves, the calculation of corresponding participation factors of these traveling modes requires that the imaginary and complex modes are also included in the expansion because they interact strongly in the region close to the damage.

The present paper answers this need and sets forth to explore the complex wavenumber solutions of the guided wave equations in high-performance composite materials. The paper is organized as follows:

First, an isotropic aluminum alloy material is chosen as a case study. The full wavenumber family (real, imaginary, complex) is calculated using the analytical formulation described in Appendix A. Then, the same wavenumber family is extracted using the semi-analytical finite element (SAFE) method which is sufficiently general to be able to handle both isotropic and anisotropic, i.e., composite, materials. The SAFE method is described in Appendix B. The reason for using the analytical method for isotropic materials is that it provides insight into the nature of the guided wave modes: symmetric vs. antisymmetric; Lamb waves vs. shear horizontal (SH) waves. This feature is used to identify modes in the SAFE results. Comparison between analytical and SAFE results for the same isotropic material allows us to conduct a convergence study of the SAFE method and to identify the minimum number of elements are needed for achieving a certain accuracy (say, 0.1%). 

Subsequently, the engineering elastic properties are modified in order to see how they influence the wavenumber trajectories in the real and imaginary planes and in the complex number volume. Note that the transverse and shear moduli of CFRP composites are much smaller than the corresponding isotropic aluminum alloy moduli. The aim is to get gradually closer to the CFRP composite properties. First, the transverse moduli are changed to understand the effect of transverse moduli on the wavenumber trajectories. Next, the shear moduli are changed to observe how the changes in shear moduli further affect the wavenumber trajectories. Finally, the wavenumber trajectories for CFRP composite are calculated. For composites, the analysis starts with a unidirectional CFRP lamina having longitudinal guided wave propagation, i.e., along the fiber direction, and the resulting wavenumber trajectories are discussed. Next, off-axis propagation and transverse propagation in composite materials are considered. Then, the wavenumber trajectories for different CFRP laminates are discussed. 

## 2. Calculation of Guided-Wave Dispersion Curves in Composite Materials

Two major approaches exist for the calculation of guided-wave dispersion curves in composites. The first approach is purely analytical and it consists of developing analytical expressions that match exactly the stress and displacement boundary conditions at the composite plate layer-by-layer interfaces and at the top and bottom faces of the plate. The second method is semi-analytical, i.e., assumes an analytical wave propagation expression as in Equation (1), but relies on the FEM approach to calculate the thickness-wise function f(y) of Equation (1). This method is usually known as SAFE, which stands for "semi-analytical finite-element". These two approaches are briefly reviewed below. 

### 2.1. Analytical Calculation of Guided Wave Dispersion Curves in Composite Materials

Many researchers have reported several methods for the propagating guided wave (real wavenumber) solution of composite materials. Global matrix method (GMM) is one of the popular methods to solve the guided wave problem in composite materials [21,22]. Knopoff [23] introduced the GMM method first. It combines stress and displacement components at the boundaries of each layer with boundary conditions. All the stress and displacement terms are assembled into one single matrix. GMM is a direct approach despite having larger global matrix size. The transfer matrix method (TMM) is another method developed by Thomson [24] and Haskell [25] to solve wave propagation in layered media. It is computationally efficient for its condensed equation system [26]. However, TMM is numerically unstable at a higher frequency [27]. Later, Wang and Rokhlin [28] proposed a reformulation of the TMM to solve the guided wave problems in a layered structure. The latter method is called the stiffness matrix method (SMM). Kamal and Giurgiutiu [29] fused SMM and TMM together to develop a stable stiffness transfer matrix method (STMM), aiming at overcoming the problem at a higher frequency.

As the literature shows, there is no such clear demonstration of imaginary and complex wavenumber solutions of composite materials. Moreover, the change in the material properties causes a change in the dispersion curve. In this paper, an attempt is made to understand the imaginary and complex wavenumber of composite materials and that could be useful for the subsequent researchers in this field. 

### 2.2. SAFE Calculation of Guided Wave Dispersion Curves in Composite Materials

**Figure 2:**
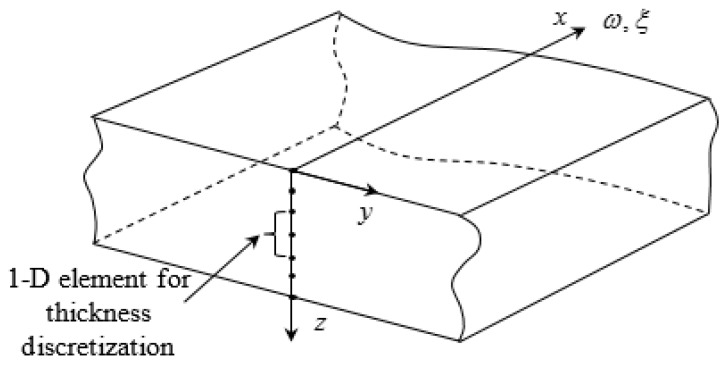
Method for guided wave solution in a plate with arbitrary material properties.

The SAFE method discretizes the cross-section of the waveguide with finite elements and uses analytical formulation along the wave propagation direction (Figure 2). The complicated variation of the wave displacement across the plate thickness is described using FEM, while the relatively simple variation in the wave-propagation direction is described analytically using complex-valued exponential functions of space and time as illustrated in Equation (1). The SAFE method combines the benefits of numerical FEM and analytical wave propagation formulations.

A SAFE method for waveguides of arbitrary cross-section was demonstrated for the first time in 1973 [30]. Since then, the SAFE approach has been used to obtain dispersion curves of isotropic and composite plates [31]. More complex waveguides, such as rods and rails [32,33,34,35] steel pipes [36], were also investigated. The main advantage of the SAFE approach is its high computational stability. For a given frequency, the SAFE formulation yields an algebraic eigenvalue problem that can be solved efficiently with existing eigenvalue extraction algorithms. The resulting eigenvalues are the wavenumbers whereas the eigenvectors are the displacement modeshapes. This process is repeated for all the frequencies of interest in a given frequency range. As in other FEM applications, a convergence study has to be considered to guarantee the simulation accuracy [37]; denser meshing is required at higher frequencies and smaller wavelengths. 

It is apparent that SAFE is a straightforward and numerically stable method for solving guided wave problems in composite structures [38,39]. The authors compared the dispersion curve of propagating wave mode of composite materials from SAFE method, unified analytical method (UAM) and commercially available DISPERSE results in an earlier publication [40]. The results from SAFE, UAM and DISPERSE agree very well. Other researchers also reported similar dispersion results for composite materials [41,42]. Hence, in this paper, the SAFE method is employed to find the real, imaginary, and complex wavenumbers of GWs in composite materials. A complete description of the SAFE method used in this study is given in Appendix B.

## 3. Stiffness Matrix

The material stiffness matrix is defined as:

(2) $$C=\left[\begin{array}{cccccc} C_{11} & C_{12} & C_{13} & 0 & 0 & 0\\ C_{21} & C_{22} & C_{23} & 0 & 0 & 0\\ C_{31} & C_{32} & C_{33} & 0 & 0 & 0\\ 0 & 0 & 0 & C_{44} & 0 & 0\\ 0 & 0 & 0 & 0 & C_{55} & 0\\ 0 & 0 & 0 & 0 & 0 & C_{66} \end{array}\right]$$

The stiffness matrix can be calculated from the compliance matrix, i.e.,

(3) $$C=S^{-1}$$

For a general orthotropic material, the compliance matrix S can be written in terms of nine engineering elastic constants $E_{1},\;E_{2},\;E_{3}$, $\nu_{12},\;\nu_{23},\;\nu_{31}$, $G_{12},\;G_{23},\;G_{31}$. As

(4) $$S=\left[\begin{array}{cccccc} \frac{1}{E_{1}} & -\frac{\nu_{12}}{E_{1}} & -\frac{\nu_{13}}{E_{1}} & 0 & 0 & 0\\ -\frac{\nu_{12}}{E_{1}} & \frac{1}{E_{2}} & -\frac{\nu_{23}}{E_{2}} & 0 & 0 & 0\\ -\frac{\nu_{13}}{E_{1}} & -\frac{\nu_{23}}{E_{2}} & \frac{1}{E_{3}} & 0 & 0 & 0\\ 0 & 0 & 0 & \frac{1}{G_{23}} & 0 & 0\\ 0 & 0 & 0 & 0 & \frac{1}{G_{31}} & 0\\ 0 & 0 & 0 & 0 & 0 & \frac{1}{G_{12}} \end{array}\right]$$

For isotropic materials, only two engineering elastic properties exist independently, E,ν; hence $E_{1}=E_{2}=E_{3}=E$, $\nu_{12}=\nu_{23}=\nu_{31}=\nu$, G12=G23=G31=G=E2(1+ν). For anisotropic composites, all six elastic constants may be independently defined. 

**Figure 3:**
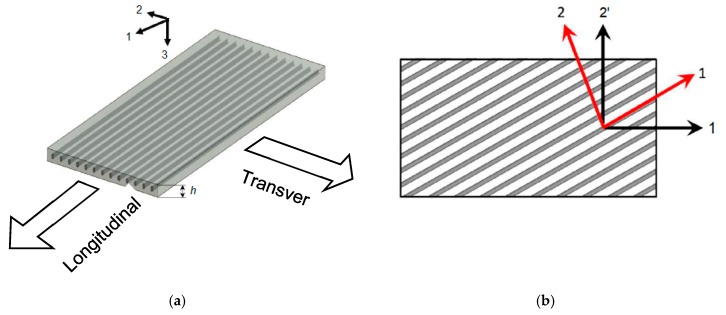
In a unidirectional fibrous composite lamina: (**a**) principal material axes; (**b**) rotated axes.

In the case of fiber-reinforced composites, such as commonly used carbon fiber reinforced polymers (CFRP), only five engineering elastic constants may exist independently due to the assumed transverse isotropy of the unidirectional prepreg ply. (Although challenged by some experimental measurements [41], this assumption is generally accepted and will maintain here). Figure 3 shows the principal directions of a fiber-reinforced composite lamina, i.e., longitudinal (L) direction along the fiber and transverse (T) across the fiber. It is apparent that this unidirectional lamina is transversely isotropic. In order to build up the stiffness matrix, a direct formulation of transversely isotropic has been used. In an experiment of the characterization the composite materials, the stiffness matrix might show orthotropic effects due to manufacturing and experimental constraints [41]. However, the symmetry in the stiffness matrix value should not differ significantly from the transversely isotropic material [43]. The objective of this paper is to build up the stiffness matrix from engineering constants and then change them gradually. The five engineering elastic properties of transversely isotropic composite lamina are $E_{L}$, $E_{T}$, $\nu_{LT}$, $G_{LT}$, $G_{23}$. The allocation of these values is done as follows: $E_{1}=E_{L}$, $E_{2}=E_{T}$, $E_{3}=E_{T}$, $\nu_{12}=\nu_{LT}$, $\nu_{13}=\nu_{LT}$, $G_{12}=G_{LT}$, $G_{23}=G_{LT}$. The in-plane Poisson ratio $\nu_{23}$ is calculated as for an isotropic material, i.e., $\nu_{23}=(E_{T}/G_{23})-1$. As a consequence, the transversely isotropic composite material stiffness matrix elements must satisfy the condition $C_{44}=(C_{22}-C_{23})/2$.

In this paper, the analysis is illustrated for two specific materials, aluminum alloy and unidirectional CFRP T300/914 composite [40]. The corresponding engineering properties are listed in Table 1 and Table 2. 

The stiffness matrices for these two materials are given below:

(5) $$C=\left[\begin{array}{cccccc} 103.7 & 51.1 & 51.1 & 0 & 0 & 0\\ 51.1 & 103.7 & 51.1 & 0 & 0 & 0\\ 51.1 & 51.1 & 103.7 & 0 & 0 & 0\\ 0 & 0 & 0 & 26.3 & 0 & 0\\ 0 & 0 & 0 & 0 & 26.3 & 0\\ 0 & 0 & 0 & 0 & 0 & 26.3 \end{array}\right]\mathrm{GPa}\;(\mathrm{aluminum}\;\mathrm{alloy})$$

(6) $$C=\left[\begin{array}{cccccc} 143.8 & 6.2 & 6.2 & 0 & 0 & 0\\ 6.2 & 13.3 & 6.5 & 0 & 0 & 0\\ 6.2 & 6.5 & 13.3 & 0 & 0 & 0\\ 0 & 0 & 0 & 3.4 & 0 & 0\\ 0 & 0 & 0 & 0 & 5.7 & 0\\ 0 & 0 & 0 & 0 & 0 & 5.7 \end{array}\right]\mathrm{GPa}\;(\mathrm{CFRP}\;\mathrm{composites})$$

**Figure 4:**
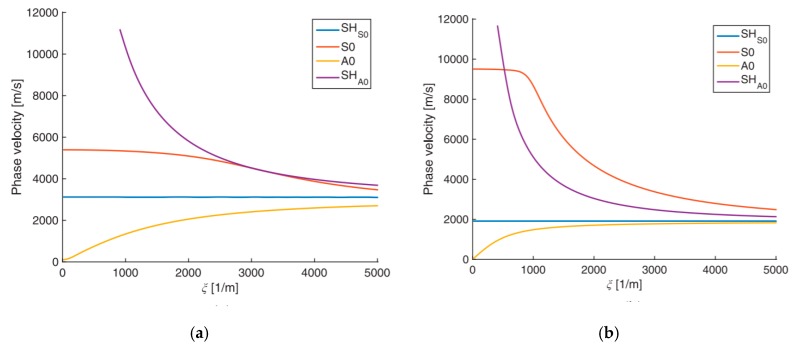
Dispersion curves (phase velocity vs wavenumber) of a 1-mm thick plate: (**a**) isotropic aluminum alloy; (**b**) orthotropic transversely isotropic CFRP [40].

Equations (5) and (6) show that CFRP composite is an order of magnitude stiffer along the fibers (L direction) than across the fibers (T directions). Another important aspect is that the shear moduli in the 23 direction are different from those in the 12 and 13 directions. These differences have a strong effect on the phase velocity-wavenumber dispersion curves of guided-wave propagation as illustrated in Figure 4 for a 1-mm thick plate [40].

It can be seen from Figure 4 that the dispersion behavior changes due to a change in the material properties. For example, the wave speed of the fundamental symmetric mode (S0) of unidirectional CFRP composites at low frequency is nearly 1.7 times higher for than of isotropic aluminum alloy. The wave speed of symmetric shear mode SH_S0_ of unidirectional CFRP composites is 1.5 times slower than isotropic aluminum alloy. Therefore, it can be concluded that the presence of anisotropy strongly modifies the real-wavenumber dispersion curves corresponding to propagating guided-wave modes. A similar effect is expected to be also observed in the dispersion curves for imaginary and complex wavenumbers, although such studies have not appeared in the literature yet, to the best of the Authors’ knowledge. 

In the subsequent analysis, the behavior of the complete wavenumber spectrum (real, imaginary, and complex) is studied starting from isotropic materials to orthotropic materials with an increasing degree of anisotropy. The reason for this gradual approach is to understand and explain how the different engineering constants affect the wavenumber trajectories in the real, imaginary, and complex domains.

Throughout the paper, a 1-mm thick plate is considered. For ease of comparison, nondimensional wavenumbers and frequencies will be used as given by:

(7) $$\bar{\Omega }=\omega d/c_{0},\;\bar{\xi }=\xi d$$

where d is half of the plate thickness. The velocity scaling factor $c_{0}$ may be chosen arbitrarily. For isotropic materials, the velocity scaling factor is equal to the shear wave speed given by $c_{S}=\sqrt{G/\rho }$, i.e., $c_{0}=c_{S}$. For orthotropic composite materials, the corresponding shear speed is the same as that of the first symmetric shear-horizontal wave SH_S0_ which has a constant wavespeed $c_{SH_{S0}}=\sqrt{C_{55}/\rho }$. Thus, for orthotropic composites, $c_{0}=\sqrt{C_{55}/\rho }$.

## 4. Frequency-Wavenumber Solution for Isotropic Materials

In isotropic plates, the GWs decouple into two separate families, (a) Lamb waves, and (b) shear-horizontal (SH) waves. Each family has symmetric and antisymmetric members. The Lamb-wave symmetric modes are denoted S0, S1, S2, …, whereas the antisymmetric Lamb modes are A0, A1, A2, … For the SH waves, the symmetric modes one denoted as SH_S0_, SH_S1_, SH_S2_, … whereas the antisymmetric modes are SH_A0_, SH_A1_, SH_A2_, … Equations (A1–A4) of Appendix A represent the separate analytical expressions for finding the wavenumbers of each of these families. The solution contains real, imaginary, and complex wavenumber roots. The wavenumber roots occur in pairs of real numbers (±ξRe), representing waves propagating in the ±x directions, as pairs of complex conjugate numbers (±ξRe±iξIm), representing damped propagating waves decaying in the ±x directions, or as pairs of purely imaginary numbers (±iξIm), representing the evanescent waves in the ±x directions.

**Figure 5:**
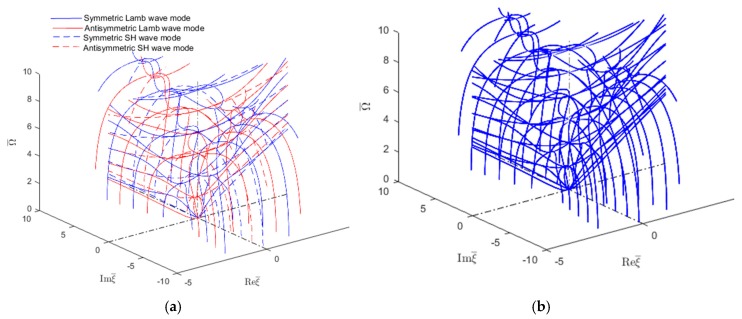
Complete wavenumber trajectories (real, imaginary and complex) for GWs in an isotropic aluminum alloy plate: (**a**) analytical method; (**b**) SAFE method.

Figure 5a shows the wavenumbers (real, imaginary, complex) trajectories in the complex wavenumber-frequency space. The symmetric Lamb wave modes are plotted with continuous blue line, the antisymmetric Lamb wave modes with continuous red line, the symmetric SH modes with dashed blue line and the antisymmetric SH modes with dashed red line. The same wavenumber roots can be also obtained with the SAFE method as shown in Figure 5b. However, the SAFE method does not distinguish between various mode types, hence the plot in Figure 5b has only one color, blue. Figure 5a,b indicate that the two methods give similar results.

Figure 5 shows that the complex wavenumbers follow continuous trajectories in the wavenumber–frequency space. One can follow some of these trajectories as they emerge from the Reξ¯−Imξ¯ plane, then climb as the frequency increases, reach a maximum, and then descend back into the Reξ¯−Imξ¯. Other wavenumber trajectories descend from above, reach a minimum, and then climb back up. A careful observation may also establish certain planes of symmetry, such as the vertical Reξ¯=0 plane, or the vertical Imξ¯=0 plane. 

**Figure 6:**
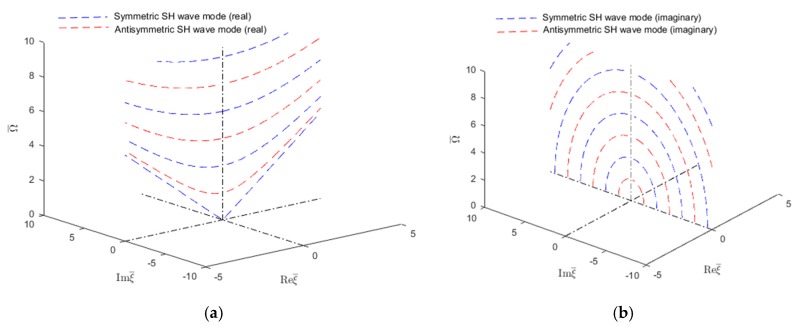
Real and imaginary SH wavenumber trajectories in an isotropic aluminum alloy plate as given by the analytical method: (**a**) real wavenumbers; (**b**) imaginary wavenumbers.

Further understanding of the wavenumber trajectories can be gained by examining certain sections in the wavenumber-frequency volume. A common section is through the two planes of symmetry Reξ¯=0 and Imξ¯=0. Figure 6 shows such plots for the SH waves. Similar plots can be drawn for the Lamb waves.

**Figure 7:**
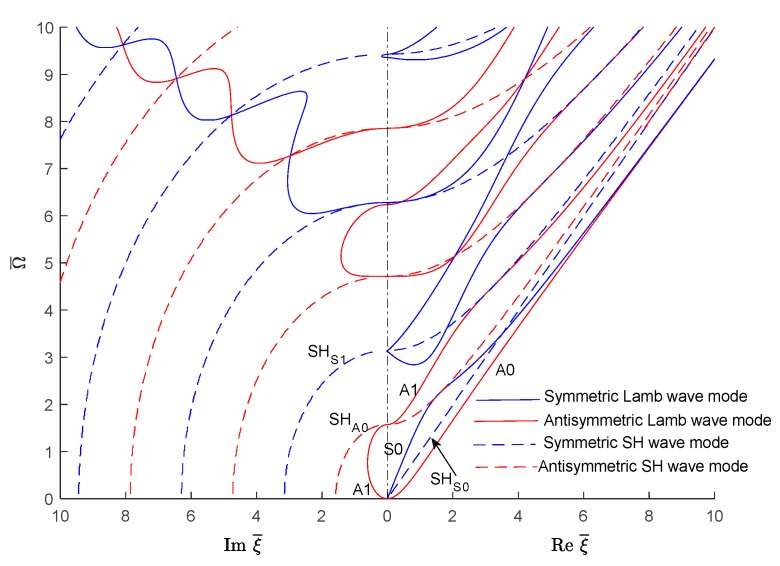
Real and imaginary wavenumber roots (combined Lamb and SH wave plot) for an isotropic aluminum alloy plate as given by the analytical method.

In order to understand the transition from evanescent to propagating waves, one should examine the locations where the wavenumbers cross the frequency vertical axis from the imaginary wavenumber plane into the real wavenumber plane. To do this, one can take only the positive sides of the two planes and join them together on the vertical frequency axis such as to have real wavenumbers plotted on right and imaginary wavenumbers plotted on the left, as shown in Figure 7 for Lamb waves and SH waves. The figure clearly shows how critical frequencies can be identified as the locations where the wavenumber trajectories cross over from the imaginary wavenumber domain into the real wavenumber domain. Figure 7 illustrates how S0 and A0 trajectories merge into a common Rayleigh-wave straight line. On the imaginary side of Figure 7, the ‘braiding’ of one symmetric and the one antisymmetric Lamb wave mode can be observed. More such braiding could also be observed at higher frequencies beyond the $\bar{\Omega }=10$ limit used in Figure 7.

**Figure 8:**
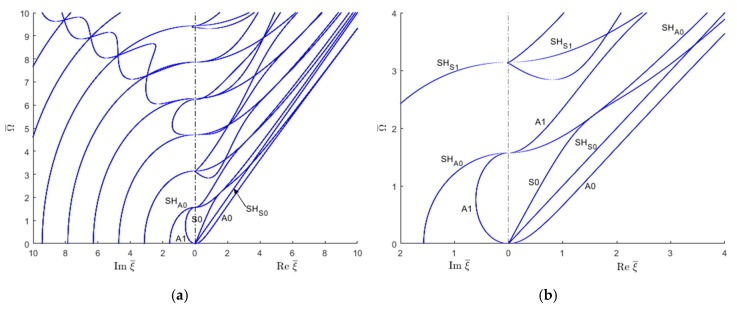
Real and imaginary wavenumber roots for an isotropic aluminum alloy plate using the SAFE method: (**a**) overall plot; (**b**) zoom in near the origin.

Similar real-imaginary wavenumber plots as shown in Figure 7 can be obtained using the SAFE method of solution (Figure 8). However, as shown in Figure 8, the SAFE method yields both the Lamb wave and SH wave modes without being able to distinguish between them a priori. In order to separate the wave modes existing in the wavenumber solution resulting from the SAFE method, one has to examine the individual modeshapes.

**Figure 9:**
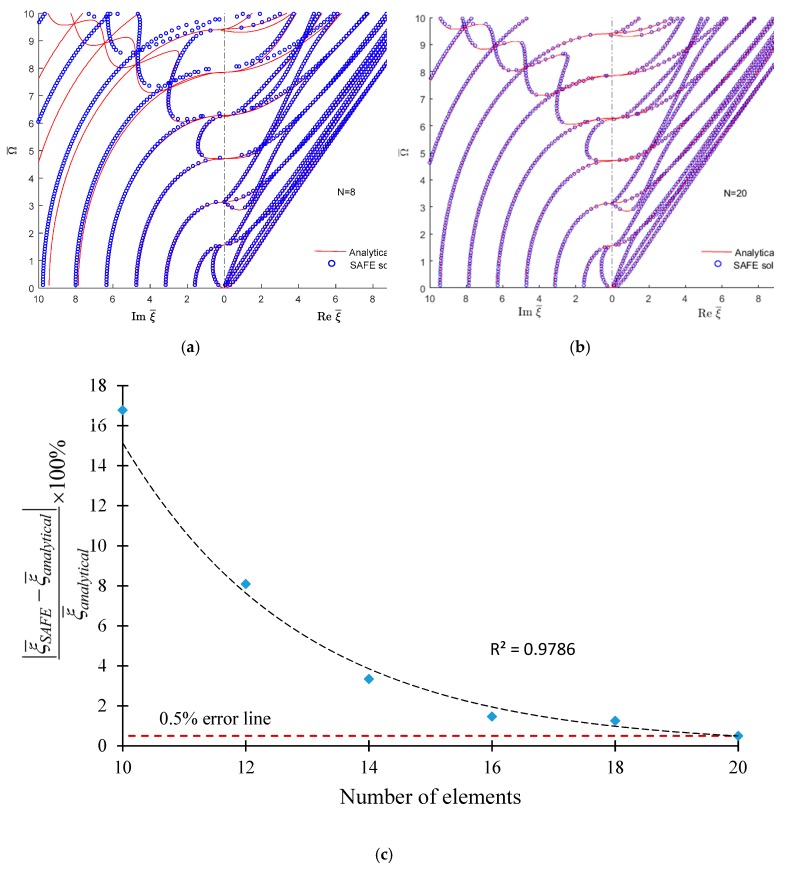
Convergence of the SAFE method in a 1-mm isotropic aluminum alloy plate showing that 0.5% accuracy in the highest wavenumber-frequency pair can be obtained with N = 20 SAFE elements across the thickness. The SAFE plots are overlapped on top of the exact analytical plots: (**a**) for N = 8, divergence from exact analytical solution at the upper left corner of the wavenumber-frequency diagram; (**b**) for N = 20, the SAFE and analytical solutions overlap everywhere; (**c**) convergence plot showing the error falling below 0.5% as N increases from N = 18 to N = 20. The trend line is a high-R exponential fit.

A convergence study of the SAFE method is also performed. Figure 9 shows that, for N = 8 FEM elements taken across the plate thickness, most of the wavenumber-frequency trajectories calculated with SAFE overlap well over the exact analytical trajectories. For the real wavenumbers, no difference can be found between the two methods. However, for high-order imaginary wavenumbers, significant differences exist as can be observed in the upper left corner of Figure 9a. These differences diminish as N increases. For N = 20, no practical difference can be observed between the SAFE and analytical results (Figure 9b). Figure 9c illustrates how the error decreases with the number of elements. The figure shows an almost exponential decay of the error, and for N = 20 the error decreases below 0.5%.

The next step is to understand the effect of various material properties on the wavenumber trajectories in the complex wavenumber-frequency domain. To achieve this aim, the isotropic material properties are modified gradually and examined is the effect of these gradual changes on the wavenumber trajectories.

## 5. Effect of Changing Material Properties

In this section, the primary goal is to understand how wavenumber trajectories are modified by incremental changes in material properties. The material properties are changed gradually from isotropic to transversely isotropic materials. The aim is to eventually get to the CFRP material properties which show very large differences from the isotropic aluminum alloy properties. For brevity, only the imaginary and real wavenumber roots are presented. However, the discussion can also be extended to the complex wavenumber roots, if needed.

### 5.1. Effect of Changing Elastic Modulus

In this section, only the elastic modulus is changed. Composite materials have a lower off-axis elastic modulus than on-axis elastic modulus ($E_{T}<<E_{L}$). Based on that concept, the transverse moduli are reduced to a lower value and $E_{2}=E_{3}=10\%E_{1}$. The density, Poisson ratio, and shear modulus are kept same as for the isotropic case. Using Equation (4) and then Equation (3), the full stiffness matrix becomes:

(8) $$C=\left[\begin{array}{cccccc} 72.3 & 3.6 & 3.6 & 0 & 0 & 0\\ 3.6 & 8.0 & 2.8 & 0 & 0 & 0\\ 3.6 & 2.8 & 8.0 & 0 & 0 & 0\\ 0 & 0 & 0 & 26.3 & 0 & 0\\ 0 & 0 & 0 & 0 & 26.3 & 0\\ 0 & 0 & 0 & 0 & 0 & 26.3 \end{array}\right]\mathrm{GPa},\;\begin{array}{l} \mathrm{low}\;\mathrm{transverse}\;\mathrm{stiffness}\\ \mathrm{high}\;\mathrm{shear}\;\mathrm{stiffness}\\ \mathrm{orthotropic}\;\mathrm{material} \end{array}$$

**Figure 10:**
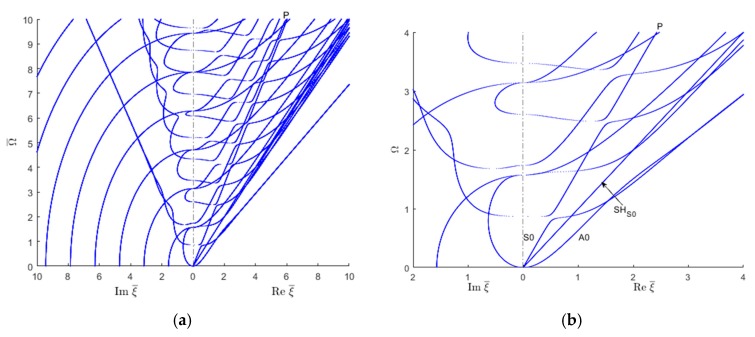
Real and imaginary wavenumber trajectories for low transverse stiffness, high shear stiffness, orthotropic material: (**a**) overall plot; (**b**) zoom in near the origin.

These material properties may not exist in practical applications, but using them helps us understand the effect of these material properties on wavenumber trajectories. Figure 10 shows the complete wavenumber solution for the modified off-axis elastic modulus. It is apparent from Figure 10 that the wavenumber solution for the shear waves remains the same as before (Figure 8) because the shear moduli $G_{12}=G_{23}=G_{31}=G$ do not change. However, changes in the trajectories of the Lamb wave wavenumbers can be observed. A downward tilt of the S0 and A0 trajectories is observed, signifying a decrease in the corresponding wavespeeds. The initial slope of the S0 line is decreased, indicating a lower quasi-axial wavespeed at low frequencies. The turn of the S0 trajectory towards the A0 trajectory occurs much sooner than in Figure 8. The S0 to A0 trajectories merge into a common Rayleigh-wave straight line sooner. The resulting Rayleigh wavenumber trajectory is tilted downward, indicating a smaller Rayleigh wavespeed.

Figure 10 shows the existence of the tendency for a straight trajectory (P line) corresponding to a quasi-pressure (i.e., dilatational) wave. This P line serves as an asymptote onto which various Lamb trajectories tend to merge on and then exit from.

Another interesting observation relates the “braiding” of symmetric-antisymmetric Lamb-wave modes on the imaginary side of the plot. This phenomenon seems to happen much sooner than in Figure 8. Also displayed is the tendency of the braided modes to form a single straight line corresponding to an evanescent Rayleigh wave mode.

### 5.2. Effect of Reducing both Transverse Elastic Modulus and Shear Modulus

In this step, the elastic modulus is kept same as in the previous step, and only the shear modulus is changed. In doing this, transversely isotropic nature of unidirectional composites is used, i.e., the elements of the stiffness matrix satisfy the relation $C_{44}=\left(C_{22}-C_{23}\right)/2$. Thus, the following procedure is applied: First, the values of $C_{22}$ and $C_{23}$ from Equation (8) are used to calculate $C_{44}=2.6\;\mathrm{GPa}$, whereas the other two shear stiffnesses are kept as they are, i.e., $C_{55}=C_{66}=26.3\;\mathrm{GPa}$. It needs to be considered also that the shear stiffness values $C_{55},C_{66}$ of composite materials are larger than the $C_{44}$ value and smaller than the $C_{22},C_{33}$ values. Thus, the stiffnesses $C_{55},C_{66}$ values are reduced to 20% of their initial values. The resulting stiffness matrix of this orthotropic transversely isotropic material with low transverse stiffness and low shear stiffness is:

(9) $$C=\left[\begin{array}{cccccc} 72.3 & 3.6 & 3.6 & 0 & 0 & 0\\ 3.6 & 8.0 & 2.8 & 0 & 0 & 0\\ 3.6 & 2.76 & 8.0 & 0 & 0 & 0\\ 0 & 0 & 0 & 2.6 & 0 & 0\\ 0 & 0 & 0 & 0 & 5.3 & 0\\ 0 & 0 & 0 & 0 & 0 & 5.3 \end{array}\right]\mathrm{GPa}\;\begin{array}{l} \mathrm{low}\;\mathrm{transverse}\;\mathrm{stiffness}\\ \mathrm{low}\;\mathrm{shear}\;\mathrm{stiffness}\\ \mathrm{orthotropic}\\ \mathrm{transversely}\;\mathrm{isotropic}\;\\ \mathrm{material} \end{array}$$

**Figure 11:**
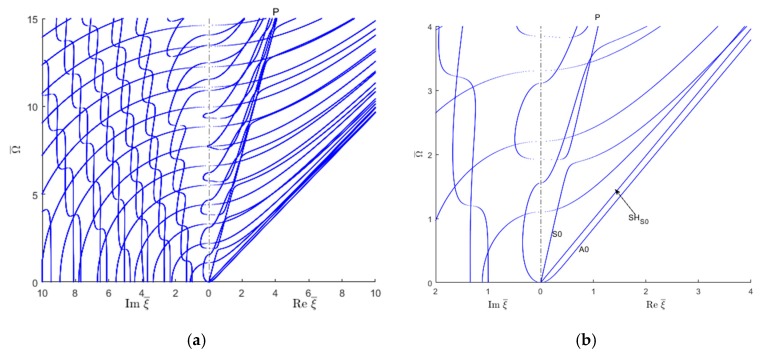
Real and imaginary wavenumber trajectories for low transverse stiffness, high shear stiffness, orthotropic transversely isotropic material: (**a**) overall plot; (**b**) zoom in near the origin.

Figure 11 shows the real and imaginary wavenumber trajectories for the transversely isotropic material. A clear change in both real and imaginary wavenumber can be observed in comparison with Figure 10. A higher frequency limit $\bar{\Omega }=15$ is chosen in order to show how the imaginary wavenumber branches connect with the real wavenumber branches.

Similar to Figure 10, it can also be observed in Figure 11 the tendency for a straight trajectory corresponding to a quasi-pressure (i.e., dilatational) wave. This P line serves as an asymptote onto which various Lamb trajectories tend to merge on and then exit from.

A major change in the behavior of the Lamb-wave imaginary wavenumber trajectories is observed when compared to the previous situation (Figure 10). As before, the braiding of the symmetric and antisymmetric Lamb wave trajectories in the imaginary side of the plot can also be observed. However, these braided trajectories seem to emanate from the horizontal axis corresponding to $\bar{\Omega }=0$. This means that an infinite number of such imaginary Lamb wave modes exists at low frequency. This is completely different from the previous cases (Figure 8 and Figure 10) when only a limited number of imaginary Lamb wave modes exists at low frequency. These symmetric and antisymmetric imaginary Lamb wave branches, which are braided at low frequency, seem to get unbraided at higher frequency, steer toward the vertical axis, and then cross over into the real domain becoming higher-order propagating Lamb waves. 

Another major change is related to SH trajectories. Many more SH modes appear in Figure 11 than in Figure 10 for the given frequency-wavenumber range. This can be attributed to the fact that the shear moduli are considerably smaller, hence resulting in a denser family of SH wavenumber trajectory in the given wavenumber range of values. 

## 6. Frequency-Wavenumber Solution for Composites

In the previous sections, an understanding is gained on how the stiffness matrix components are likely to affect the real and imaginary wavenumber trajectories. Now, the practical situation of a CFRP unidirectional composite lamina is considered which is an orthotropic transversely isotropic material with properties given in Equation (6), i.e.,

(10) $$\left[C\right]=\left[\begin{array}{cccccc} 143.8 & 6.2 & 6.2 & 0 & 0 & 0\\ 6.2 & 8.03 & 6.2 & 0 & 0 & 0\\ 6.2 & 6.5 & 6.5 & 0 & 0 & 0\\ 0 & 0 & 0 & 3.4 & 0 & 0\\ 0 & 0 & 0 & 0 & 5.7 & 0\\ 0 & 0 & 0 & 0 & 0 & 5.7 \end{array}\right]\mathrm{GPa}$$

As before, a 1-mm thick plate is considered. The wave propagation is assumed to be in the fiber direction $x_{1}$.

### 6.1. Frequency-Wavenumber Solution for Unidirectional Composites

**Figure 12:**
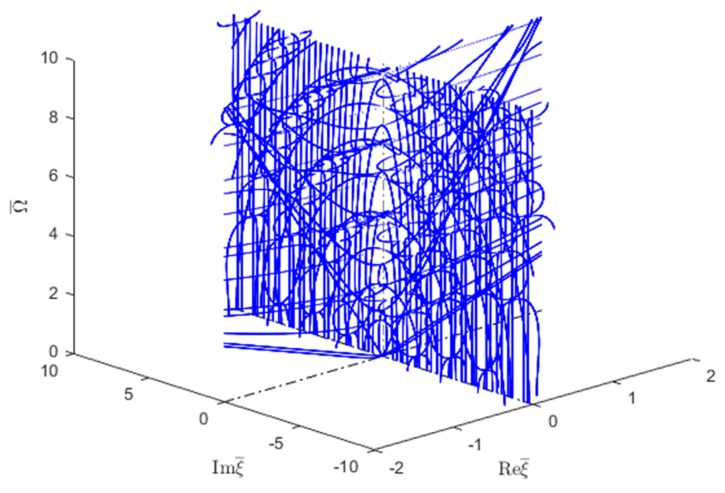
wavenumber solution of GWs for unidirectional CFRP composite.

**Figure 13:**
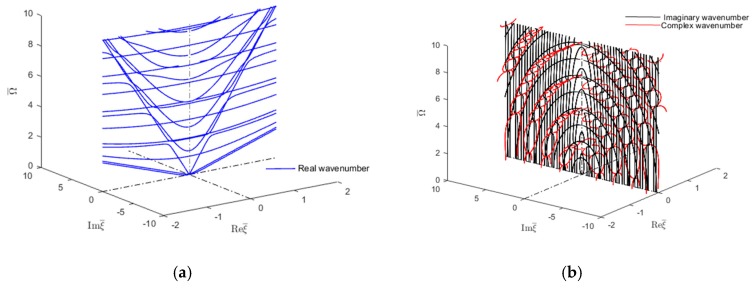
Wavenumber trajectories of GWs in unidirectional CFRP composites: (**a**) real wavenumbers; (**b**) imaginary and complex wavenumbers.

The complete wavenumber solution for unidirectional composites contains a complicated network or trajectories as illustrated in Figure 12. To facilitate understanding, this plot is separated into two plots, as shown in Figure 13. Here, Figure 13a presents only the real wavenumber trajectories, i.e., those contained in the vertical plane Imξ¯=0, whereas Figure 13b presents the imaginary and complex wavenumber trajectories. In Figure 13b, the imaginary wavenumber trajectories are contained in the vertical plane Reξ¯=0, whereas the complex wavenumbers are 3D trajectories in-and-out of the vertical plane Reξ¯=0. The complex wavenumbers can be written in a general form as:

(11) $$\xi =\xi_{R}+i\xi_{I}$$

where $\xi_{R}$ and $\xi_{I}$ are the real and imaginary parts of the complex wavenumber ξ. Note that Equation (11) refers to physical wavenumbers obtained from the nondimensional wavenumber using the scaling relation given in Equation (7). Recall now the generic wave field of Equation (1), and let the wavenumber be a complex quantity as defined by Equation (11) i.e.,

(12) $$f\left(y\right)e^{i(\xi x-\omega t)}=f\left(y\right)e^{i((\xi_{R}+i\xi_{I})x-\omega t)}=f\left(y\right)e^{-\xi_{I}x}e^{i(\xi_{R}x-\omega t)}\;$$

For real wavenumber solution only, ${\bar{\xi }}_{I}=0$. Therefore, the wave field equation becomes:

(13) $$f\left(y\right)e^{i(\xi x-\omega t)}=f\left(y\right)e^{i(\xi_{R}x-\omega t)}\;\in x\geq 0$$

The real wavenumber solutions of the guided wave represent wave modes that are harmonic in both space and time. The modes corresponding to the real wavenumber solution propagate and carry power along the propagation direction. These modes are propagating modes.

For the imaginary wavenumber solution only, ${\bar{\xi }}_{R}=0$. Therefore, the wave field equation becomes:

(14) $$f\left(y\right)e^{i(\xi x-\omega t)}=f\left(y\right)e^{-\xi_{I}x}e^{-i\omega t}\in x\geq 0$$

The amplitude of this particular type of wave mode exponentially decays with space and does not carry power along the propagation direction. These modes are called evanescent modes because they represent non-propagating local vibration that decays rapidly away from the source. The motion of these modes stays trapped in space near the source. 

The complex wavenumbers appear in conjugate pairs. Therefore, the function of Equation (12) becomes:

(15) $$f\left(y\right)e^{i((\xi_{R}+i\xi_{I})x-\omega t)}+f\left(y\right)e^{i((-\xi_{R}+i\xi_{I})x-\omega t)}=2f\left(y\right)e^{-\xi_{I}x}\cos\xi_{R}xe^{-i\omega t}\in x\geq 0$$

The amplitude of the wave modes corresponding to complex wavenumber follows exponentially decaying cosine function in space while being harmonic in time. Therefore, these modes exist near the source as rapidly decaying waves. The complex mode pairs do not carry any power flow along the propagation direction. 

### 6.2. Separation of Guided Wave Modes in CFRP Composites

**Figure 14:**
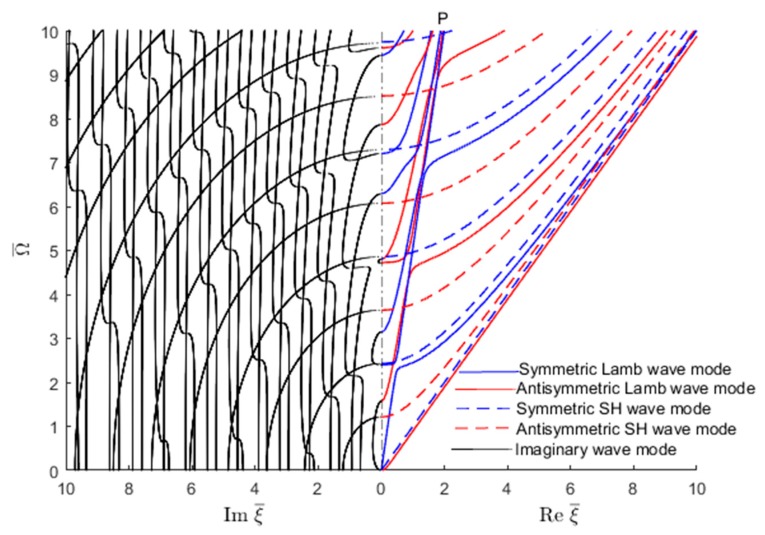
Real and imaginary wavenumber solution of GWs for CFRP composites.

The wavenumber solution can be sorted in various guided wave modes based on the mode shape. Figure 14 shows the usual side-by-side plot of imaginary and real wavenumbers. The separated symmetric and antisymmetric quasi-Lamb and quasi-SH modes based on the displacement modeshapes are presented in a later section. The propagating wave modes are identified based on the displacement mode shape (Symmetric antisymmetric of Lamb wave or SH wave) e.g., the displacement in the propagating direction $\left(u_{x}\right)$ or thickness direction $\left(u_{z}\right)$ would be more dominant than that in the transverse direction displacement $\left(u_{y}\right)$ for Lamb wave types, whereas the transverse direction displacement $\left(u_{x}\right)$ would be dominant for the SH type wave mode. For the symmetric Lamb wave mode, the wave propagation direction displacement $\left(u_{x}\right)$ is the same for the top and bottom of the plate but opposite for the anti-symmetric wave mode. For the symmetric SH wave mode, the transverse direction displacement $\left(u_{y}\right)$ is the same for the top and bottom of the plate, whereas for the anti-symmetric wave mode it is opposite. For imaginary and complex wave modes, the mode shapes are sorted based on the significant components (e.g., real or imaginary part of the displacement mode shape which follows a similar trend as the displacement mode shape of the propagating wave). Figure 14 shows how the propagating quasi-Lamb waves and quasi-SH waves represented by real wavenumbers connect with the corresponding evanescent waves represented by imaginary wavenumbers.

Similar to Figure 10 and Figure 11, it can also be observed in Figure 14 the tendency for a straight trajectory (P line) resembling a quasi-pressure (i.e., dilatational) wave. This P line serves as an asymptote onto which various Lamb trajectories tend to merge on and then exit from.

To facilitate the understanding further, this plot is separated into symmetric and antisymmetric modes, and then into quasi-Lamb and quasi-SH wave modes. Then, these modes are compared with the corresponding isotropic modes. 

#### 6.2.1. Symmetric Quasi-Lamb Modes

**Figure 15:**
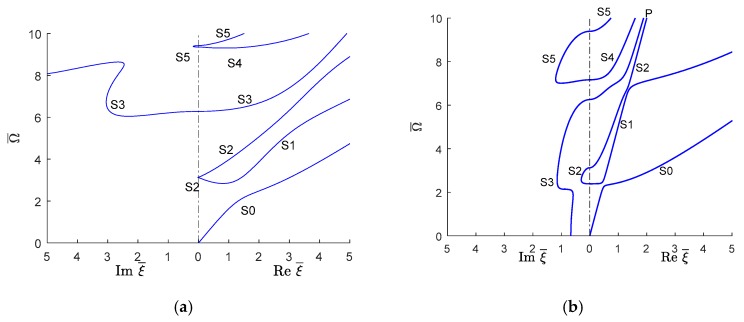
Real (propagating) and imaginary (evanescent) wavenumber solution of symmetric Lamb wave type GWs for (**a**) isotropic aluminum alloy; (**b**) unidirectional CFRP composite.

Figure 15 shows the comparison of symmetric Lamb wave modes between an isotropic aluminum alloy and unidirectional CFRP composite. In each plot, the imaginary (evanescent) wavenumber trajectories are presented on the left whereas the real (propagating) wavenumber trajectories are on the right. The imaginary trajectories connect with the real trajectories at the vertical frequency axis corresponding to $\bar{\xi }=0$. It should be noted that, at the beginning, i.e., at low frequencies, the fundamental symmetric (S0) wave mode for CFRP composites has a much steeper slope than isotropic aluminum alloy. Therefore, it can be concluded that the low-frequency phase velocity of S0 mode is much higher in unidirectional CFRP composites than in the isotropic aluminum alloy. A similar phenomenon can also be observed for the higher order symmetric modes. Therefore, in practical applications, the wave speed of symmetric modes along the fiber direction of a CFRP composite is expected to be much higher than in an isotropic aluminum alloy. As one goes to higher wavenumber values, one notices that the quasi-S modes tend towards a common P line on which they dwell for a while and, subsequently, depart from it with a much smaller slope, i.e., lower phase velocity. This transition from high slope to low slope happens at lower wavenumbers and is much quicker than for the isotropic aluminum alloy.

Additional observation of Figure 15 reveals several major differences in the behavior of symmetric evanescent wave modes. For the isotropic aluminum alloy, no imaginary wavenumber exists up to $\bar{\Omega }=3.118$ as shown in Figure 15a. Then, there is a small region where the imaginary symmetric wave mode S2 appears briefly; this is followed by a region in which no imaginary symmetric wave mode exists up to $\bar{\Omega }=6.05$. Beyond this limit, one can find always an imaginary symmetric wave mode (e.g., S3) for a given value of $\bar{\Omega }$. However, as shown in Figure 15b for unidirectional CFRP composites, an imaginary wavenumber always exists at any given $\bar{\Omega }$ value. 

Another notable difference in the imaginary wave mode is that, for the isotropic aluminum alloy, there is only a small region where the S2 $\left(\bar{\Omega }\approx 3.118\;\mathrm{to}\;3.142\right)$ and S5 $\left(\bar{\Omega }\approx 9.355\;\mathrm{to}\;9.425\right)$ imaginary mode exists, whereas in CFRP composites, the S2 and S5 imaginary mode range is bigger than for the isotropic aluminum alloy. For CFRP composites, the S2 imaginary mode started at $\bar{\Omega }\approx 2.39$ and is converted to a real wavenumber after crossing the zero-wavenumber axis at $\bar{\Omega }\approx 3.13$. The imaginary S5 mode starts at $\bar{\Omega }\approx 7.17$ and converts to the real part at $\bar{\Omega }\approx 9.4$. The imaginary S3 mode converts to the real S3 mode at $\bar{\Omega }\approx 6.26$. For the isotropic aluminum alloy, the S3 imaginary modes go up whereas for unidirectional CFRP composites, the S3 mode goes down to zero wavenumbers. 

#### 6.2.2. Antisymmetric Quasi-Lamb Modes

**Figure 16:**
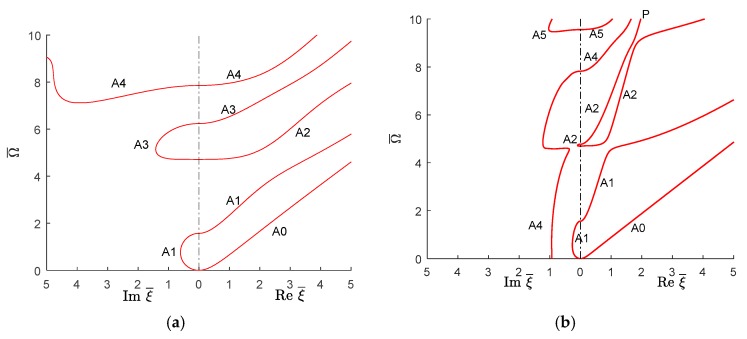
Real (propagating) and imaginary (evanescent) wavenumber solution of antisymmetric Lamb wave type GWs for (**a**) isotropic aluminum alloy; (**b**) unidirectional CFRP composite.

Figure 16 shows the real (propagating) and imaginary (evanescent) wavenumber trajectories for antisymmetric quasi-Lamb wave modes in the isotropic aluminum alloy and unidirectional CFRP composite. It is apparent in Figure 16 that the fundamental antisymmetric quasi-Lamb mode A0 of unidirectional CFRP composites shows similar behavior as in the isotropic aluminum alloy. However, the higher order antisymmetric modes show significant differences. The higher order propagating modes of unidirectional CFRP composites start with a steeper slope than the isotropic ones. Therefore, the initial phase velocity of higher antisymmetric propagating wave is higher than in the isotropic aluminum alloy. However, at higher wavenumber values, the quasi-A modes tend towards a common P line on which they dwell for a while and, subsequently, depart from it with a much smaller slope, i.e., lower phase velocity. This transition from high slope to low slope happens at lower wavenumbers and is much quicker than for the isotropic aluminum alloy.

Similar to the symmetric imaginary wavenumbers, there are some regions where no antisymmetric imaginary modes exist in isotropic aluminum alloy. For example in the region of $\bar{\Omega }\approx 1.571$ to $\bar{\Omega }\approx 4.712$ and $\bar{\Omega }\approx 6.237$ to $\bar{\Omega }\approx 7.12$, there is no antisymmetric imaginary mode for isotropic aluminum alloy. However, after $\bar{\Omega }\approx 7.12$ there is always an antisymmetric imaginary mode exist for isotropic aluminum alloy. For unidirectional CFRP composite, this situation is different and one always finds an imaginary antisymmetric quasi-Lamb mode for any given $\bar{\Omega }$ value as illustrated in Figure 16b. For unidirectional CFRP composite, the quasi-A1 antisymmetric imaginary mode starts at $\bar{\Omega }=0$ and converts to real quasi-A1 mode at $\bar{\Omega }\approx 1.56$. There is a small region where the imaginary A3 mode exists ($\bar{\Omega }\approx 4.69$ to $\bar{\Omega }\approx 4.77$). The imaginary A4 mode converts to real A4 mode at $\bar{\Omega }\approx 7.82$. For isotropic aluminum alloy, the A4 imaginary mode goes up, whereas this mode goes down to zero wavenumbers for unidirectional CFRP composites.

#### 6.2.3. Quasi-SH Modes

**Figure 17:**
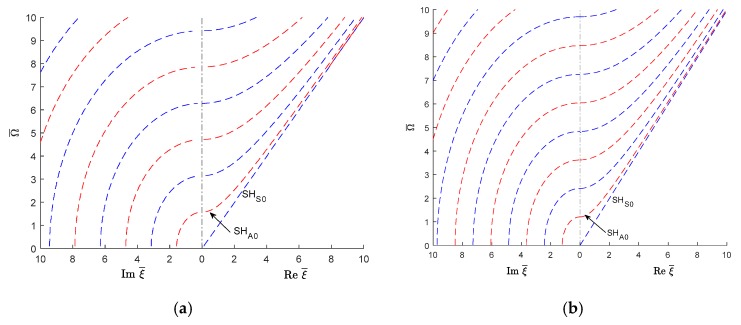
Real (propagating) and imaginary (evanescent) wavenumber solution of shear horizontal (SH) wave type GWs for (**a**) isotropic aluminum alloy; (**b**) unidirectional CFRP composite.

Figure 17 shows the real (propagating) and imaginary (evanescent) wavenumber trajectories of symmetric and antisymmetric SH waves for the isotropic aluminum alloy and unidirectional CFRP composite. The symmetric and antisymmetric SH wave modes appear alternatively. The important difference between isotropic and unidirectional CFRP is that the number of shear wave modes for unidirectional CFRP is higher than for the isotropic aluminum alloy for the given frequency-wavenumber range. This is may be due to the difference in shear wave speed. The unidirectional CFRP has lower shear wave speed ($c_{SH_{S0}}\approx 1910\;m/s$) than the isotropic one ($c_{s}\approx 3120\;m/s$). The SH wave motion depends on the shear moduli $G_{23}$ and $G_{12}$. These values for unidirectional CFRP composites are 3.4 and 5.7 GPa, whereas for the isotropic aluminum alloy, the value is 26.3 GPa.

#### 6.2.4. Wavenumber Trajectories in the 3D Complex Space

**Figure 18:**
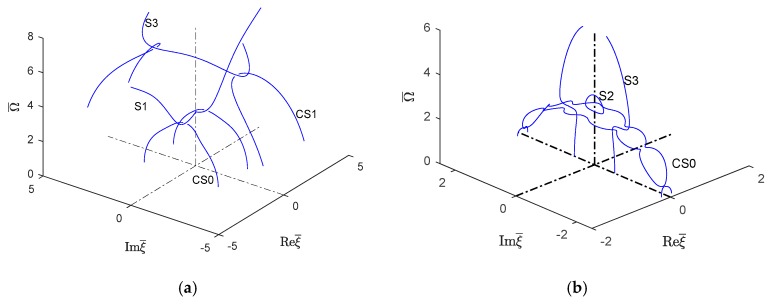
Real (propagating) and imaginary (evanescent) and complex wavenumber solution for the first few symmetric modes of Lamb wave type GWs for (**a**) isotropic material and (**b**) unidirectional CFRP composite.

**Figure 19:**
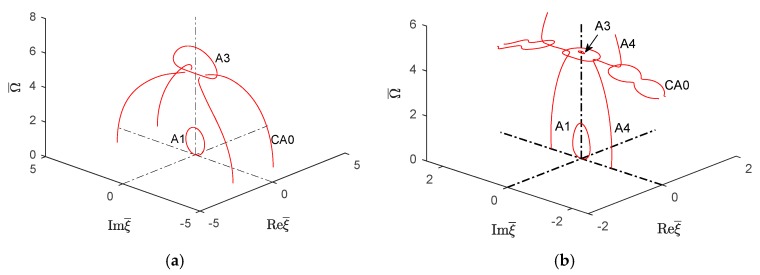
Imaginary (evanescent) and complex wavenumber solution for the first few antisymmetric modes of Lamb wave type GWs for (**a**) isotropic material and (**b**) unidirectional CFRP composite.

Following the wavenumber trajectories in the 3D complex space allowed to understand the nature of different wavenumber branches as they move from real to imaginary and then complex number behavior and connect with each other. This is illustrated in Figure 18 and Figure 19 for symmetric and antisymmetric quasi-Lamb modes. In each figure, the isotropic aluminum alloy is compared with the unidirectional CFRP composite. The real part of both symmetric and antisymmetric complex wavenumbers is smaller for unidirectional CFRP composites than for the isotropic aluminum alloy. Therefore, the CFRP complex wavenumbers plots tend to be somehow "squashed" into the vertical imaginary plane, whereas the aluminum alloy plots are more extended in the real-wavenumber directions. It can also be observed that the 3D complex wavenumber branches tend to connect with real or imaginary wavenumber branches, this being true for both isotropic aluminum alloy and unidirectional CFRP materials.

Figure 18a,b compare a few complex Lamb modes in the isotropic aluminum alloy with the corresponding modes in the unidirectional CFRP composite. It can be seen that, for the isotropic aluminum alloy, the first symmetric complex mode (CS0) is connected with the symmetric propagating wave mode (S1); however, the next complex wave mode CS1 is connected with the imaginary symmetric evanescent S3 wave mode, whereas for CFRP unidirectional composites, Figure 18b shows the complex wave mode CS0 having two branches around the evanescent modes S2 and S3. The behavior of higher order symmetric complex modes can be seen in Figure 13b which shows the complex branch separating into some elliptical shapes and connected with the imaginary symmetric modes. 

Figure 19a shows that, for the isotropic aluminum alloy, the first antisymmetric complex mode CA0 is connected with the antisymmetric evanescent wave mode A3, whereas for CFRP unidirectional composites, Figure 19b shows that the complex mode CA0 has different branches connected with the evanescent antisymmetric mode A4. The antisymmetric evanescent mode A3 is at the center of the elliptical branch of the complex mode. The subsequent higher order symmetric and antisymmetric wave mode follows a similar pattern to CA0-A3 as shown in Figure 13b. 

### 6.3. Effect of Propagation Direction

So far, propagation in a unidirectional CFRP composite along the fiber direction has been investigated. This situation is usually referred to as on-axis propagation, or 0° propagation. However, wave propagation can also happen in an off-axis direction, say at 45°, or transverse to the fiber, i.e., at 90°. Hence, it is important to study and understand the effect of propagation direction on wavenumber trajectories. For any angle θ, the stiffness matrix of Equation (10) can be rotated to yield the rotated stiffness matrix:

(16) $$C_{\theta }=T^{-1}C\;T^{-t}$$

where:

(17) $$T=\left[\begin{array}{cccccc} \cos^{2}\theta & \sin^{2}\theta & 0 & 0 & 0 & 2\sin\theta \cos\theta \\ \sin^{2}\theta & \cos^{2}\theta & 0 & 0 & 0 & -2\sin\theta \cos\theta \\ 0 & 0 & 1 & 0 & 0 & 0\\ 0 & 0 & 0 & \cos\theta & -\sin\theta & 0\\ 0 & 0 & 0 & \sin\theta & \cos\theta & 0\\ -\sin\theta \cos\theta & \sin\theta \cos\theta & 0 & 0 & 0 & \cos^{2}\theta -\sin^{2}\theta \end{array}\right],\;\left(\begin{array}{c} 3D\;\\ \mathrm{rotation}\;\\ \mathrm{matrix} \end{array}\right)$$

Here, guided wave propagation in 45° and 90° directions in unidirectional CFRP composite plates are studied. After rotation, the stiffness matrices become:

(18) $${\left[C\right]}_{45}=\left[\begin{array}{cccccc} 48.1 & 36.7 & 6.4 & 0 & 0 & 32.6\\ 36.7 & 48.1 & 6.2 & 0 & 0 & 32.6\\ 6.4 & 6.5 & 13.3 & 0 & 0 & -0.15\\ 0 & 0 & 0 & 4.6 & 1.2 & 0\\ 0 & 0 & 0 & 1.2 & 4.6 & 0\\ 32.6 & 32.6 & -0.15 & 0 & 0 & 36.2 \end{array}\right]\mathrm{GPa}$$

(19) $${\left[C\right]}_{90}=\left[\begin{array}{cccccc} 13.3 & 6.2 & 6.5 & 0 & 0 & 0\\ 6.2 & 143.8 & 6.2 & 0 & 0 & 0\\ 6.5 & 6.2 & 13.3 & 0 & 0 & 0\\ 0 & 0 & 0 & 5.7 & 0 & 0\\ 0 & 0 & 0 & 0 & 3.4 & 0\\ 0 & 0 & 0 & 0 & 0 & 5.7 \end{array}\right]\mathrm{GPa}$$

**Figure 20:**
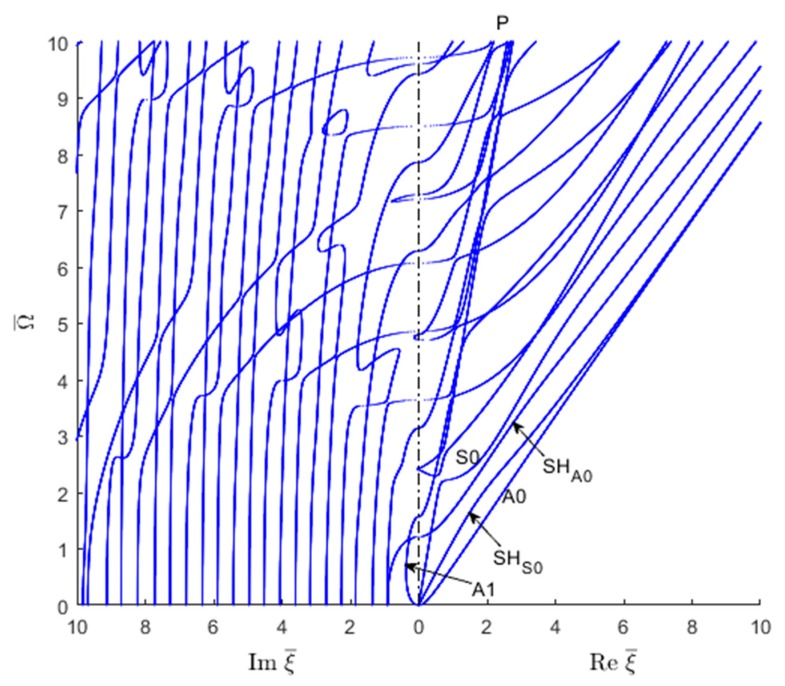
Complex wavenumber trajectories for 45-degree off-axis wave propagation in a 1-mm laminated unidirectional CFRP composite plate (wave propagation direction at 45-degree with respect to fiber direction).

**Figure 21:**
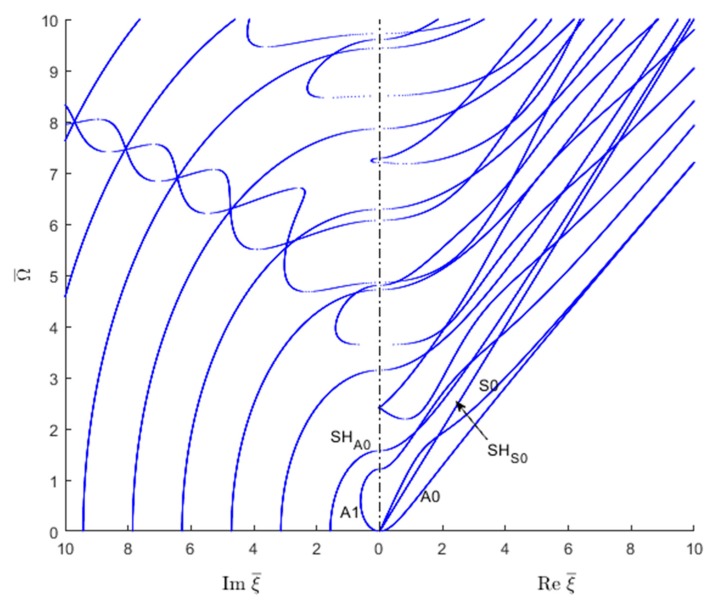
Complex wavenumber trajectories for 90-degree wave propagation in a 1-mm laminated unidirectional CFRP composite plate (wave propagation direction is perpendicular to the fiber direction).

The wavenumber trajectories for the 45° and 90° directions are given in Figure 20 and Figure 21, respectively. In understanding the behavior of the wavenumber trajectories, one could use the ratio $C_{11}/C_{55}$ as a guide. From Equations (10), (18) and (19), one can find that $C_{11}/C_{55}$ ratio is 25.2, 10.4, and 3.6 for 0°, 45°, and 90° directions, respectively, whereas, in the isotropic aluminum alloy, the $C_{11}/C_{55}$ value is 3.9. Therefore, the wavenumber solution for the 45° wave propagation direction (Figure 20) seems closer to that in the 0° direction (i.e., high $C_{11}/C_{55}$ value) and seems to have a degree of complexity similar to wavenumber solution for 0° direction. The important characteristic in Figure 20 is that the wave speed of the fundamental symmetric SH mode is not constant anymore but rather it changes with the frequency. A tendency of quasi-SH_S0_ mode merging with the quasi-A0 mode is also observed. It can also notice that the higher order SH modes are distorted, which is different from what is observed for 0° wave propagation. The behavior of the wavenumber trajectories for the quasi-SH modes can be explained as a coupling effect between different shear moduli as shown in Equation (18). Some distortions are also observed in the evanescent wave modes (Figure 20) and that may be due to the coupling effect of different elastic moduli.

For the 90° wave propagation direction in unidirectional CFRP, the $C_{11}/C_{55}$ value is closer to the isotropic one. Therefore, the wavenumber solution for 90° wave propagation direction (Figure 21) shows a similar pattern as the isotropic aluminum alloy despite having different wavenumber trajectories for the various wave modes. For example, the braiding of the symmetric and antisymmetric quasi-Lamb modes observed in the imaginary side of the plot (left pane) is very similar to that observed for the isotropic aluminum alloy (Figure 8). Higher order SH modes behave similar to the isotropic aluminum alloy ones. The similarity can also be observed in propagating and evanescent wave mode though the frequency-wavenumber pair values are different.

### 6.4. Real, Imaginary, and Complex Modes in Laminated Composites

**Figure 22:**
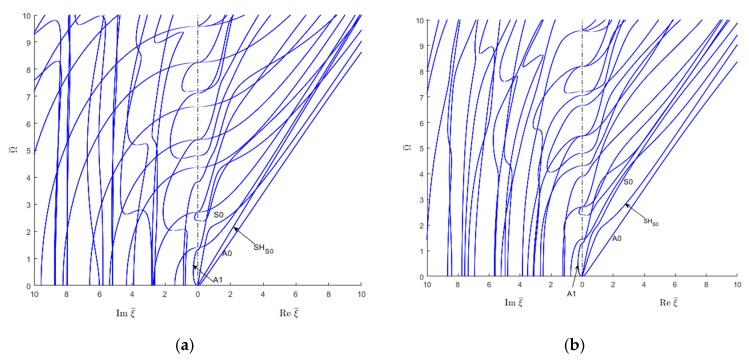
Complex wavenumber trajectories for 1-mm laminated CFRP composite plates: (**a**) 8-layer cross ply [0/90/0/90]_S_; (**b**) 8-layer quasi-isotropic [0/+45/−45/90]_S._

Laminated composites are made up of several layers of unidirectional lamina oriented at various angles according to the staking sequence. Quite common stacking sequences are angle ply, cross-ply, and quasi-isotropic. The SAFE method is used to extract the wavenumber roots for two stacking sequences, cross-ply [0/90]_2S_ and quasi-isotropic [0/+45/−45/90]_S_ as presented in Figure 22. By comparing the wavenumber trajectories for cross-ply laminate (Figure 22a) with those for the unidirectional composite (Figure 14), it can be seen that the wave speed of the first SH mode remains constant whereas for higher SH modes, the wavenumber trajectories resemble those for unidirectional composite. One also notices that the quasi-S0 and quasi-A0 modes have a tendency to merge at high frequency-wavenumber values.

For quasi-isotropic composites (Figure 22b), the wave speed of the first SH mode is not constant. That is may be due to the coupling between different shear moduli contributing to the SH wave speed. It can also be illustrated from Figure 22b that the higher order SH modes for quasi-isotropic are distorted in comparison with unidirectional ones. Some changes in the evanescent modes in comparison with unidirectional case are observed for both cross-ply and quasi-isotropic composites.

### 6.5. Convergence of the Safe Method in Composite Materials

**Figure 23:**
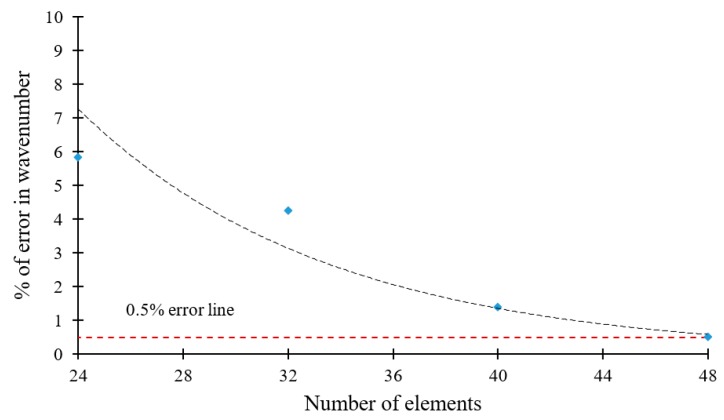
Convergence of SAFE method in the 1-mm quasi-isotropic [0/+45/−45/90]_S_ CFRP composite plate: 0.5% accuracy in the highest wavenumber-frequency pair of the evanescent kind can be obtained with N = 48 SAFE elements across the thickness. The trend line is an exponential fit.

A convergence study of the SAFE method when applied to composites is conducted. Figure 23 shows how error decrease in a 1-mm quasi-isotropic [0/+45/−45/90]_S_ CFRP composite plate as the number of SAFE elements increases across the thickness. An error less than 0.5% in the highest wavenumber-frequency pair of the evanescent kind is obtained for N = 48 SAFE elements across the thickness. The trend line of the error decrease seems to be an exponential fit. The number of elements and corresponding error for various other CFRP composite layups are presented in Table 3.

## 7. Conclusions and Future Work

The main goal of this paper is to study for the first time, the behavior of composite material wavenumber trajectories in the complex wavenumber-frequency space. The knowledge of the complex wavenumber trajectories is the essential ingredient for attempting a normal mode expansion solution to the wave-damage interaction in composite structures. 

The main conclusions of the present study are:

1. SAFE is a robust and reliable method for determining the complex wavenumber solution as confirmed by the fact that the SAFE results match well with the exact analytical solution for an isotropic aluminum alloy.
2. A material change study moving gradually from an isotropic aluminum alloy to CFRP composites was performed. The material change study shows that reducing the transverse and shear moduli moves the wavenumber solution towards one similar to that of composite material.
3. The comparisons of wavenumber trajectories between the isotropic aluminum alloy and CFRP composites show that the fundamental antisymmetric wave mode of unidirectional CFRP composites looks similar to that of isotropic materials. However, higher order antisymmetric wave modes for CFRP composite materials start showing discrepancy from isotropic materials.
4. For isotropic materials, there are some frequency regions where the imaginary Lamb wave modes do not exist. However, for CFRP composites, imaginary quasi Lamb wave modes always exist at any given frequency.
5. For isotropic materials, the trajectory of the first symmetric complex wave mode is connected with the trajectory of the second propagating wave mode, whereas the subsequent symmetric complex trajectories are connected with the symmetric imaginary trajectories. In composites, the complex symmetric trajectories are always connected with the symmetric imaginary trajectories. The antisymmetric complex trajectories are always connected with the imaginary antisymmetric trajectories for both unidirectional CFRP and isotropic materials.
6. The results for off-axis, transverse, cross-ply and quasi-isotropic laminates show that there is a significance change in SH wave trajectories due to presence of ±45 ply in composite laminates. The wavenumber trajectory behavior of such composite laminates is governed by $C_{11}/C_{55}$ ratio.
7. The convergence of the SAFE method in the isotropic aluminum alloy requires N = 20 thickness-wise elements to achieve <0.5% error. In CFRP composites, the SAFE convergence depends on the laminate layup. However, N = 48 ensures <1% error in the highest evanescent wavenumber in all the CFRP composites considered in this study.

Future work will address the stress-continuity between composite layers. At present, the stress mode shapes resulting from SAFE analysis do not satisfy the stress-continuity condition between composite layers and hence cannot be directly used in a normal mode expansion approach that uses power orthogonality between the displacement and stress components of the wave modes. Future work will investigate other approaches (e.g., analytical or hybrid analytical-SAFE) for calculating of the correct stress mode shapes for composite guided waves.

## Figures and Tables

**Table 1:** Properties of aluminum alloy.

Elastic Modulus, E	70.0 GPa
Poisson ratio, ν	0.33
Density, ρ	2700 kg/m^3^

**Table 2:** Properties of CFRP T300/914 composites.

Longitudinal Elastic Modulus, $E_{L}$	140.0 GPa
Transverse elastic modulus, $E_{T}$	10.05 GPa
Shear modulus, $G_{LT}$	5.70 GPa
Shear modulus, $G_{23}$	3.4 GPa
Poisson ratio, $\nu_{LT}$	0.313
Density, ρ	1560 kg/m^3^

**Table 3:** Number of SAFE elements across the thickness to obtain at least 1.0% convergence for the highest wavenumber-frequency pair in evanescent mode in various CFRP composite layups.

Laminate Type	Number of Elements in Current Step, N	% Error in Wavenumber Value $\frac{\xi_{\mathrm{Precious}\;\mathrm{step}}-\xi_{\mathrm{current}\;\mathrm{step}}}{\xi_{\mathrm{Precious}\;\mathrm{step}}}\times 100\%$	No. of Elements Increment in Each Step
Unidirectional CFRP [0]_8_	40	0.6	2
Off-axis CFRP [45]_8_	30	0.9	2
Transverse CFRP [90]_8_	30	0.2	2
Cross-ply CFRP [0/90]_2s_	40	0.2	8
Quasi-isotropic CFRP [0/+45/−45/90]_s_	48	0.5	8

## Nomenclature

**Abbreviations**	**Description**
CFRP	Carbon fiber reinforced plastic
SAFE	Semi-analytical finite element
SHM	Structural health monitoring
GWs	Guided waves
FEM	Finite element method
GMM	Global matrix method (GMM)
TMM	Transfer matrix method
SMM	Stiffness matrix method
STMM	Stiffness transfer matrix method
SH	Shear-horizontal
LWS	Symmetric Lamb wave
LWA	Anti-symmetric Lamb wave
SHWS	Symmetric shear horizontal wave
SHWA	Anti-symmetric shear horizontal wave
3D	Three dimensional

**Parameters**	**Description**
S0	First symmetric wave mode
S1	Second symmetric wave mode
S2	Third symmetric wave mode
A0	First anti-symmetric wave mode
A1	Second anti-symmetric wave mode
A2	Third anti-symmetric wave mode
SH_S0_	First symmetric SH wave mode
SH_S1_	Second symmetric SH wave mode
SH_S1_	Third symmetric SH wave mode
SH_A1_	First anti-symmetric SH wave mode
SH_A2_	Second anti-symmetric SH wave mode
SH_A3_	Third anti-symmetric SH wave mode
N	Number of elements
ξ	Wavenumber
ω	Frequency
c	Wave speed
E	Elastic constant
G	Shear modulus
υ	Poisson’s ratio
C	Stiffness matrix
S	Compliance matrix
ρ	Density
$\bar{\Omega }$	Non dimensional frequency
$\bar{\xi }$	Non-dimensional wavenumber
d	Plate half thickness
$c_{0},c_{S},c_{SH_{S0}}$	First symmetric shear wave speed
$c_{P}$	Pressure wave speed
θ	Fiber orientation angle
$u_{x}$	Propagating direction displacement
$u_{y}$	Thickness direction displacement
$u_{z}$	Transverse direction displacement

## Appendix A. Analytical Method for Extracting Isotropic Plate Wavenumbers

The analytical solution for GWs in isotropic plates is described by four uncoupled characteristic equations, two for Lamb waves and two for shear horizontal (SH) waves. The pair of characteristic equations for the Lamb waves is also known as the Raleigh-Lamb equation (a single RL equation with a +/− sign in it). In this work, the characteristic equations in terms of nondimensional variables $\bar{\xi }$, $\bar{\Omega }$ are used, i.e.,

(A1) $$f_{LWS}\left(\bar{\xi },\;\bar{\Omega }\right)={\left({\bar{\xi }}^{2}-{\bar{\eta }}_{S}^{2}\right)}^{2}\cos{\bar{\eta }}_{P}\sin{\bar{\eta }}_{S}+4{\bar{\eta }}_{P}{\bar{\eta }}_{S}{\bar{\xi }}^{2}\sin{\bar{\eta }}_{P}\cos{\bar{\eta }}_{S}=0\;(\mathrm{Sym}.\;\mathrm{Lamb}\;\mathrm{waves})$$

(A2) $$f_{LWA}\left(\bar{\xi },\;\bar{\Omega }\right)={\left({\bar{\xi }}^{2}-{\bar{\eta }}_{S}^{2}\right)}^{2}\sin{\bar{\eta }}_{P}\cos{\bar{\eta }}_{S}+4{\bar{\eta }}_{P}{\bar{\eta }}_{S}{\bar{\xi }}^{2}\cos{\bar{\eta }}_{P}\sin{\bar{\eta }}_{S}=0\;(\mathrm{Antisym}.\;\mathrm{Lamb}\;\mathrm{waves})$$

(A3) $$f_{SHS}\left(\bar{\xi },\;\bar{\Omega }\right)=\sin{\bar{\eta }}_{S}=0\;(\mathrm{symmetric}\;\mathrm{SH}\;\mathrm{waves})$$

(A4) $$f_{SHA}\left(\bar{\xi },\;\bar{\Omega }\right)=\cos{\bar{\eta }}_{S}=0\;(\mathrm{antisymmetric}\;\mathrm{SH}\;\mathrm{waves})$$

where ${\bar{\eta }}_{S}$, ${\bar{\eta }}_{S}$ are functions of $\bar{\xi }$, $\bar{\Omega }$, i.e.,

(A5) $${\bar{\eta }}_{P}=\sqrt{\frac{{\bar{\Omega }}^{2}}{\kappa^{2}}-{\bar{\xi }}^{2}},\;{\bar{\eta }}_{S}=\sqrt{{\bar{\Omega }}^{2}-{\bar{\xi }}^{2}}$$

(A6) $$c_{P}=\sqrt{\frac{(1-\nu )E}{(1+\nu )(1-2\nu )\rho }},\;c_{S}=\sqrt{\frac{E}{2(1+\nu )\rho }},\;\kappa =c_{P}/c_{S}$$

The characteristic Equations (A1–A4) are solved numerically through a complex root searching routine [7].

## Appendix B. SAFE Method for Extracting Wavenumbers in An Arbitrary-Material Plate

This section presents the semi-analytical finite element (SAFE) method for guided wave solution in composite structures. It should be noted that the presented solution is also valid for an isotropic structure. The SAFE method discretizes the cross-section of the structure with finite elements and uses analytical formulation along the wave propagation direction. The waves propagate along the x direction with wavenumber ξ at angular frequency ω. The cross-section lies in the y−z plane.

The guided-wave displacement field is assumed to be of the form:

(A7) $$u\left(x,y,z,t\right)=\left[\begin{array}{c} u_{x}\left(x,y,z,t\right)\\ u_{y}\left(x,y,z,t\right)\\ u_{z}\left(x,y,z,t\right) \end{array}\right]=\left[\begin{array}{c} U_{x}\left(y,z\right)\\ U_{y}\left(y,z\right)\\ U_{z}\left(y,z\right) \end{array}\right]e^{i\left(\xi x-\omega t\right)}$$

The harmonic displacement, stress, and strain field components at each point of the waveguide are expressed by:

(A8) $$\begin{array}{l} u={\left[\begin{array}{ccc} u_{x} & u_{y} & u_{z} \end{array}\right]}^{T}\\ \sigma ={\left[\begin{array}{cccccc} \sigma_{x} & \sigma_{y} & \sigma_{z} & \sigma_{yz} & \sigma_{xz} & \sigma_{xy} \end{array}\right]}^{T}\\ \varepsilon ={\left[\begin{array}{cccccc} \varepsilon_{x} & \varepsilon_{y} & \varepsilon_{z} & \varepsilon_{yz} & \varepsilon_{xz} & \varepsilon_{xy} \end{array}\right]}^{T} \end{array}$$

The constitutive equation is given by:

(A9) σ=Cε

where C is the stiffness matrix. The stress-displacement relation can be written as:

(A10) $$\varepsilon =\left[L_{x}\frac{\partial }{\partial x}+L_{y}\frac{\partial }{\partial y}+L_{z}\frac{\partial }{\partial z}\right]u$$

where:

(A11) $$L_{x}=\left[\begin{array}{ccc} 1 & 0 & 0\\ 0 & 0 & 0\\ 0 & 0 & 0\\ 0 & 0 & 0\\ 0 & 0 & 1\\ 0 & 1 & 0 \end{array}\right],\;L_{y}=\left[\begin{array}{ccc} 0 & 0 & 0\\ 0 & 1 & 0\\ 0 & 0 & 0\\ 0 & 0 & 1\\ 0 & 0 & 0\\ 1 & 0 & 0 \end{array}\right],\;L_{z}=\left[\begin{array}{ccc} 0 & 0 & 0\\ 0 & 0 & 0\\ 0 & 0 & 1\\ 0 & 1 & 0\\ 1 & 0 & 0\\ 0 & 0 & 0 \end{array}\right]$$

The governing equation is obtained by inserting the kinetic and potential energies into Hamilton’s equation:

(A12) $$\delta H=\int_{t_{1}}^{t_{2}}\delta \left(\Phi -K\right)\;dt=0$$

where Φ is the strain energy and K is the kinetic energy. The strain energy and kinetic energy are given by:

(A13) $$\Phi =\frac{1}{2}\int_{V}\varepsilon^{T}C\varepsilon \;dV\;K=\frac{1}{2}\int_{V}{\dot{u}}^{T}\rho \dot{u}dV$$

where V is the volume, ρ is the material density. After integrating by parts, Equation (A12) can be written as:

(A14) $$\int_{t_{1}}^{t_{2}}\left[\int_{V}\delta \left(\varepsilon^{T}\right)C\varepsilon dV+\int_{V}\delta \left(u^{T}\right)\rho \ddot{u}\;dV\right]\;dt=0$$

Finite element discretization in the thickness direction is done by 1-D quadratic isoparametric element. The displacements over the element domain are expressed in terms of the shape functions $N_{k}\left(z\right)$ and the unknown nodal displacements $U_{kx}$, $U_{ky}$, and $U_{kz}$ of a node k in x, y, and z direction is :

(A15) $$u^{\left(e\right)}\left(x,z,t\right)={\left[\begin{array}{c} \sum_{k=1}^{N}N_{k}\left(z\right)U_{kx}\\ \sum_{k=1}^{N}N_{k}\left(z\right)U_{ky}\\ \sum_{k=1}^{N}N_{k}\left(z\right)U_{kz} \end{array}\right]}^{\left(e\right)}e^{i\left(\xi x-\omega t\right)}=N\left(z\right)q^{\left(e\right)}e^{i\left(\xi x-\omega t\right)}$$

(A16) $$\mathrm{where},\;N\left(z\right)=\left[\begin{array}{ccccccccc} N_{1} & & & N_{2} & & & N_{3} & & \\ & N_{1} & & & N_{2} & & & N_{3} & \\ & & N_{1} & & & N_{2} & & & N_{3} \end{array}\right]$$

(A17) $$q^{\left(e\right)}={\left[\begin{array}{ccccccccc} U_{1x} & U_{1y} & U_{1z} & U_{2x} & U_{2y} & U_{2z} & U_{3x} & U_{3y} & U_{3z} \end{array}\right]}^{T}$$

The symbol (e) means the elemental expression. The strain vector can also be expressed in terms of nodal displacement and shape functions.

(A18) $$\varepsilon^{\left(e\right)}=\left[L_{x}\frac{\partial }{\partial x}+L_{y}\frac{\partial }{\partial y}+L_{y}\frac{\partial }{\partial y}\right]N\left(z\right)q^{\left(e\right)}e^{i\left(\xi x-\omega t\right)}=\left(B_{1}+i\xi B_{2}\right)q^{\left(e\right)}e^{i\left(\xi x-\omega t\right)}$$

where:

(A19) $$B_{1}=L_{y}\frac{\partial N}{\partial y}+L_{z}\frac{\partial N}{\partial z};\;B_{2}=L_{x}N$$

In general, a Jacobian matrix is used to convert the derivatives from the global coordinate to the local isoparametric coordinate. For the 1-D quadratic element, the Jacobian becomes a constant, and the derivative in global coordinate can be expressed as:

(A20) $$\frac{dN}{dz}=\frac{dN}{d\zeta }\frac{d\zeta }{dz}=\frac{1}{J}\frac{dN}{d\zeta }$$

where the Jacobian, according to Equation (A20), is evaluated as:

(A21) $$J=\frac{dz}{d\zeta }=\left[\begin{array}{ccc} \zeta -\frac{1}{2} & -2\zeta & \zeta +\frac{1}{2} \end{array}\right]\left[\begin{array}{c} z_{1}\\ z_{2}\\ z_{3} \end{array}\right]$$

Let $n_{e}$ is the total number of elements along the plate thickness, the discretized of Equation (A12) is:

(A22) $$\int_{t_{1}}^{t_{2}}\left\{\overset{n_{e}}{\underset{e=1}{\cup }}\left[\int_{V_{e}}\delta \left(\varepsilon^{\left(e\right)T}\right)C_{e}\varepsilon^{\left(e\right)}\;dV_{e}+\int_{V_{e}}\delta \left(u^{\left(e\right)T}\right)\rho_{e}{\ddot{u}}^{\left(e\right)}\;dV_{e}\right]\right\}\;dt=0$$

where $C_{e}$ and $\rho_{e}$ are the stiffness matrix and density of the corresponding element. For multilayer laminated composites, these material properties need to be defined for the elements in each layer. The symbol ∪ denotes the assembling procedure.

The substitution of Equation (A18) into the strain energy term of Equation (A22) yields:

(A23) $$\int_{V_{e}}\delta \left(\varepsilon^{\left(e\right)T}\right){\tilde{C}}_{e}\varepsilon^{\left(e\right)}\;dV_{e}=\delta q^{\left(e\right)T}\int_{\Omega_{e}}\left[B_{1}^{T}C_{e}B_{1}-i\xi B_{2}^{T}C_{e}B_{1}+i\xi B_{1}^{T}C_{e}B_{2}+\xi^{2}B_{2}^{T}C_{e}B_{2}\right]\;d\Omega_{e}q^{\left(e\right)}$$

where $\Omega_{e}$ is the elemental domain.

Substitution of Equation (A15) into the kinetic energy term of Equation (A22) yields:

(A24) $$\int_{V_{e}}\delta \left(u^{\left(e\right)T}\right)\rho_{e}{\ddot{u}}^{\left(e\right)}\;dV_{e}=-\omega^{2}\delta q^{\left(e\right)T}\int_{\Omega_{e}}N^{T}\rho_{e}N\;d\Omega_{e}q^{\left(e\right)}$$

Substitution of Equations (A23) and (A24) into Equation (A22) yields:

(A25) $$\int_{t_{1}}^{t_{2}}\left\{\overset{n_{e}}{\underset{e=1}{\cup }}\delta q^{\left(e\right)}\left[k_{1}^{\left(e\right)}+i\xi k_{2}^{\left(e\right)}+\xi^{2}k_{3}^{\left(e\right)}-\omega^{2}m^{\left(e\right)}\right]q^{\left(e\right)}\right\}\;dt=0$$

where:

(A26) $$\begin{array}{l} k_{1}^{\left(e\right)}=\int_{\Omega_{e}}\left[B_{1}^{T}C_{e}B_{1}\right]\;d\Omega_{e}\;k_{2}^{\left(e\right)}=\int_{\Omega_{e}}\left[B_{1}^{T}C_{e}B_{2}-B_{2}^{T}C_{e}B_{1}\right]\;d\Omega_{e}\\ k_{3}^{\left(e\right)}=\int_{\Omega_{e}}\left[B_{2}^{T}C_{e}B_{2}\right]\;d\Omega_{e}\;m^{\left(e\right)}=\int_{\Omega_{e}}\left[N^{T}\rho_{e}N\right]\;d\Omega_{e} \end{array}$$

Upon applying the standard finite element assembling procedure, Equation (A25) becomes:

(A27) $$\int_{t_{1}}^{t_{2}}\left\{\delta U^{T}\left[K_{1}+i\xi K_{2}+\xi^{2}K_{3}-\omega^{2}M\right]U\right\}\;dt=0$$

where U is the global vector of unknown nodal displacements, and:

(A28) $$K_{1}=\overset{n_{e}}{\underset{e=1}{\cup }}k_{1}^{\left(e\right)}\;K_{2}=\overset{n_{e}}{\underset{e=1}{\cup }}k_{2}^{\left(e\right)}\;K_{3}=\overset{n_{e}}{\underset{e=1}{\cup }}k_{3}^{\left(e\right)}\;M=\overset{n_{e}}{\underset{e=1}{\cup }}m^{\left(e\right)}$$

Since Equation (A27) is true for any arbitrary δU, the following homogeneous equation describing the wave displacement across the thickness is obtained:

(A29) $$\left[K_{1}+i\xi K_{2}+\xi^{2}K_{3}-\omega^{2}M\right]U=0$$

Equation (A29) can be written in an equivalent form as:

(A30) $$\left(\left[\begin{array}{cc} 0 & K_{1}-\omega^{2}M\\ K_{1}-\omega^{2}M & iK_{2} \end{array}\right]-\xi \left[\begin{array}{cc} K_{1}-\omega^{2}M & 0\\ 0 & -K_{3} \end{array}\right]\right)\left[\begin{array}{c} U\\ \xi U \end{array}\right]=0$$

Equation (A30) is an algebraic eigenvalue problem. If M is the dimension of the vector U, then, at each frequency ω, Equation (A30) yields 2M eigenvalues ξ and 2M eigenvectors U. However, the eigenvalues occur in pairs: the real eigenvalues occur as positive/negative pairs, appropriate for forward and backward propagation. For the imaginary and complex eigenvalues, complex-conjugate pairing also appears.
